# Steroid-Induced Diabetic Ketoacidosis: A Case Report and Review of the Literature

**DOI:** 10.7759/cureus.24372

**Published:** 2022-04-22

**Authors:** Megan M Cavataio, Clifford D Packer

**Affiliations:** 1 Internal Medicine, Case Western Reserve University School of Medicine, Cleveland, USA

**Keywords:** keto acidosis, steroid-induced, diabetic ketoacidosis (dka), ketosis prone diabetes, ketosis-prone type 2 diabetes, steroid-induced diabetes

## Abstract

It has been well documented that corticosteroid treatment can precipitate hyperglycemia and may lead to new diagnoses of type 2 diabetes mellitus. However, steroid-induced diabetic ketoacidosis (DKA) has rarely been reported in the literature. We report the case of an obese 73-year-old man with no known history of diabetes mellitus who presented with DKA after two months of treatment with high-dose steroids. Our patient’s presentation and clinical course were consistent with ketosis-prone type 2 diabetes (KPDM-2). A literature review revealed three other reports of patients with steroid-induced DKA, two of whom also had clinical and biochemical features that were consistent with KPDM-2. We postulate that high-dose steroid treatment can trigger DKA in a subgroup of obese, middle-aged patients with risk factors for KPDM-2. Physicians should suspect steroid-induced KPDM-2 in obese patients who present with new-onset DKA after initiation of steroid treatment.

## Introduction

Hyperglycemia has been reported to occur in as many as 64-71% of patients treated with corticosteroids [1-2]. The hyperglycemic effects of steroids have been shown to worsen previously diagnosed diabetes, and can also trigger the development of new diabetes in 1.5-27% of patients placed on steroid therapy [3-4]. However, steroid-induced diabetic ketoacidosis (DKA) has rarely been reported. We report a case of steroid-induced DKA in an obese 73-year-old man with clinical and biochemical findings that were consistent with ketosis-prone type 2 diabetes (KPDM-2).

DKA is most frequently associated with type 1 diabetes mellitus; it can also occur in the setting of latent autoimmune diabetes in adults (LADA) and in ketosis-prone type 2 diabetes (KPDM-2). Patients with KPDM-2 are typically middle-aged, male, obese, and have other dysmetabolic traits such as hypertension or hypercholesterolemia. They present with DKA and always require insulin treatment initially, but once the glucose toxicity has resolved they can often be weaned gradually off insulin and treated with oral diabetic medications and lifestyle modifications [5-6]. They do not have autoantibodies against pancreatic beta cells and often have normalization of C-peptide levels after treatment of glucose toxicity, which differentiates this syndrome from type 1 diabetes and LADA. The cellular mechanism of KPDM-2 involves metabolic defects in the mitochondria that inhibit the oxidation of ketones leading to ketoacidosis. Additionally, extremely high serum glucose levels and associated oxidative stress lead to glucose toxicity, with decreased insulin secretion from the pancreas and increased insulin resistance primarily in the liver, skeletal muscle, and adipose tissue, which contributes to the development of further hyperglycemia [7-8]. Mechanisms for steroid-induced diabetes include increased glucose production via hepatic gluconeogenesis and glycogenolysis, and increased peripheral insulin resistance at the adipose and muscle level via inhibition of GLUT4 translocation [9-10].

## Case presentation

A 73-year-old Caucasian man with hypertension, obesity, coronary artery disease, heart failure with reduced ejection fraction, chronic kidney disease stage 3 secondary to IgA nephropathy, monoclonal gammopathy of undetermined significance, and hypercholesterolemia was diagnosed with hypersensitivity pneumonitis and started on prednisone 40 mg daily. Two months later, he presented to the hospital with polydipsia, polyuria, generalized weakness, and unintentional 25-pound weight loss over six weeks. On physical examination, vital signs were blood pressure of 130/59, pulse rate of 82, respiratory rate of 21, and temperature of 97.3 °F; breath sounds were mildly diminished, heart sounds were normal with no murmur or gallop, and the abdomen was soft and non-tender; the neurologic exam was non-focal. The body mass index (BMI) was 33.5. Initial laboratory results were significant for hyperglycemia (glucose 723 mg/dL) with anion gap metabolic acidosis, hyperkalemia, and acute kidney injury (see Table 1).

**Table 1:** Initial laboratory results at time of hospitalization

Glucose	Hemoglobin A1c	Potassium	BUN	Creatinine	Anion gap	Serum ketones	Urine ketones	pH	PCO2	PO2	HCO3-	Anti-insulin antibody	Anti-islet cell antibody	Anti-glutamic acid decarboxylate antibody (GAD)
723 mg/dL	13.80%	5.8 mEq/L	60 mg/dl	2.4 mg/dl	20	‘Moderate’	‘Moderate’	7.3	31	128	16.3	Negative	Negative	Negative

The patient had no prior personal or family history of diabetes; a baseline hemoglobin A1c was not available, but random (non-fasting) glucose from two to three months prior to admission had ranged from 124-168 mg/dL. Medications on admission were prednisone 20 mg daily (the dose had been tapered down slowly from 40 mg daily over the two months prior to admission), atorvastatin, losartan, metoprolol, omeprazole, aspirin, clopidogrel, and evolocumab. 

He was treated initially in the intensive care unit for diabetic ketoacidosis with intravenous fluids, potassium, and an insulin drip. The anion gap closed to 11 by 12 hours post-admission, and the patient’s blood glucose levels eventually stabilized to between 100-200 mg/dl. The acute kidney injury and hyperkalemia resolved with the treatment of ketoacidosis. Retinal examination by an optometrist revealed no evidence of diabetic retinopathy, and the patient did not develop signs or symptoms of peripheral neuropathy. There was stable, low-grade albuminuria with urine microalbumin/creatinine ratio ranging between 26-142 for six years before hospital admission, which was attributed to the patient’s biopsy-proven IgA nephropathy.

He was discharged on 44 units daily of basal and prandial insulin, and empagliflozin and metformin were added to his regimen as an outpatient. He gradually recovered from hypersensitivity pneumonitis, and the prednisone was tapered off and finally discontinued three months after discharge. Six months after discharge, his total insulin requirement had decreased to 15 units daily and the hemoglobin A1c was down to 6.0%.

## Discussion

Our patient’s clinical and laboratory presentation met the criteria for DKA, which occurred after two months on a course of high-dose prednisone for hypersensitivity pneumonitis. He did not have a prior diagnosis of diabetes mellitus; the temporal connection with his high-dose steroid treatment and lack of any other precipitating cause suggest that his DKA was precipitated by the steroids. In regard to the underlying cause of his DKA, tests for autoantibodies against pancreatic beta cells in our patient were negative, so type 1 diabetes and LADA were unlikely. The negative GAD antibody result and his status as an elderly obese man with underlying hypertension and hyperlipidemia whose first indication of diabetes was DKA all support a diagnosis of steroid-induced KPDM-2. The successful weaning down of his insulin dose to 15 units daily by six months after discharge with excellent glycemic control (hemoglobin A1c 6.0%) also fits with the diagnosis of KPDM-2.

Steroid-induced DKA is a rare phenomenon [11]. A PubMed literature search revealed only three other cases of steroid-induced DKA in adults (Table 2) [12-14].

**Table 2:** Comparison of patients with known steroid-induced DKA (N/A= data not available)

Case Report	Age	Sex	BMI (kg/m2)	Reason for steroid treatment	Prior DM II Dx	Post-treatment C-peptide	Insulin auto-antibody	pH/HCO3	Anion gap	Clinical outcome
Our patient	73	M	33.5	Hypersensitivity pneumonitis	No	N/A	Negative	7.31/17.1	20	Insulin dose reduced from 44 to 15 units/day at six months
Alakkas et al. [12]	53	F	35	Immune thrombocytopenic purpura	No	N/A	N/A	7.1/3.2	27	Off insulin at six months, on metformin
Tiwari et al. [13]	55	F	48	Lumbar disc herniation	Yes	3.5 nmol/L	Negative	7.1/2.0	29	N/A
Rahman et al. [14]	59	F	N/A	Cerebral edema	Yes	N/A	N/A	7.28/15	"High"	N/A

In one case, a 53-year-old woman developed diabetic ketoacidosis after being diagnosed with immune thrombocytopenic purpura and three months of treatment with methylprednisolone. This patient, like ours, had no prior diagnosis of diabetes and was also obese with a BMI of 35 kg/m2 [12]. Another case report describes a 55-year-old woman with a history of well-controlled type 2 diabetes and a BMI of 48 kg/m2 who presented with DKA one week after she was started on prednisone 40 mg BID for lumbar disc herniation [13]. Finally, a 59-year-old woman with type 2 diabetes, hypertension, and a high-grade glioma presented with cerebral edema for which she received a total dose of 24 mg of IV dexamethasone over five hours; during surgery, she was found to have an anion gap metabolic acidosis and hyperglycemia and was diagnosed with DKA [14]. In all of these cases, there was a strong temporal association between the initiation of steroid treatment and the development of DKA, with no other precipitating factors for DKA such as infection, stroke, antipsychotic drugs, heavy alcohol consumption, or substance abuse. Based on the available data, three of the four patients were obese, two patients who were tested had negative insulin auto-antibodies, and one was found to have a normal post-treatment C-peptide level. Of the two patients who had a long-term follow-up, one had been weaned off insulin at six months, and the other (our patient) had had his insulin dose reduced by more than 60%. Obesity, negative insulin auto-antibodies, post-treatment normalization of C-peptide levels, and the ability to wean off insulin are all key features of KPDM-2. We postulate that high-dose steroid treatment (either long- or short-term) can trigger DKA in a subgroup of obese, middle-aged to elderly patients with risk factors for KPDM-2.

## Conclusions

We present a rare case of steroid-induced DKA in a patient whose clinical and biochemical presentation was consistent with KPDM-2. Steroid-induced DKA is rarely reported, but it could be more common than the handful of published cases would suggest. Physicians should suspect steroid-induced KPDM-2 in obese patients who present with new-onset DKA after initiation of steroid treatment. In addition, patients with obesity and other dysmetabolic risk factors who require steroid treatment should be carefully monitored for symptoms of DKA, a potentially life-threatening condition.
